# Characteristics and outcomes of patients admitted to intensive care units in Uganda: a descriptive nationwide multicentre prospective study

**DOI:** 10.1038/s41598-024-59031-5

**Published:** 2024-04-30

**Authors:** Patience Atumanya, Peter. K. Agaba, John Mukisa, Jane Nakibuuka, Arthur Kwizera, Cornelius Sendagire

**Affiliations:** 1 Uganda Cancer Institute, Kampala, Uganda; 2 Makerere University, Kampala, Uganda; 3 Mulago Hospital, Kampala, Uganda; 4 Uganda Heart Institute, Kampala, Uganda; 5 Intensive Care Medicine, D’or Institute for Research and Education, Rio de Janeiro, Brazil

**Keywords:** Low-income country, Intensive care unit, Patient characteristics, Risk factors, Mortality, Risk factors, Epidemiology, Outcomes research

## Abstract

Intensive care unit (ICU) mortality rates have decreased over time. However, in low-and lower-middle income countries (LMICs), there remains an excess ICU mortality with limited understanding of patient characteristics, treatments, and outcomes from small single centre studies. We aimed therefore, to describe the characteristics, therapies and outcomes of patients admitted to all intensive care units in Uganda. A nationwide prospective observational study including all patients admitted Uganda’s ICUs with available daily charts was conducted from 8th January 2018 to 1st April 2018. Socio-demographics and clinical characteristics including worst vital signs in the first 24 h of admission were recorded with calculation of the National Early Warning Score (NEWS-2) and quick Sequential Organ Function Assessment (qSOFA) score. ICU interventions were recorded during the ICU stay and patients were followed up to 28 days in ICU. The primary outcome was 28 day ICU mortality. Three-hundred fifty-one patients were analysed with mean age 39 (24.1) years, 205 (58.4%) males with 197 (56%) surgical admissions. The commonest indication for ICU admission was postoperative care (42.9%), 214 (61%) had at least one comorbidity, with hypertension 104 (48.6%) most prevalent and 35 (10%) HIV positive. The 28 day ICU mortality was 90/351 (25.6%) with a median ICU stay of 3 (1–7) days. The highest probability of death occurred during the first 10 days with more non-survivors receiving mechanical ventilation (80% vs 34%; p < 0.001), sedation/paralysis (70% vs 50%; p < 0.001), inotropic/vasopressor support (56.7% vs 22.2%; p < 0.001) and renal replacement therapy (14.4% vs 4.2%; p < 0.001). Independent predictors of ICU mortality included mechanical ventilation (HR 3.34, 95% CI 1.48–7.52), sedation/paralysis (HR 2.68, 95% CI 1.39–5.16), inotropes/vasopressor (HR 3.17,95% CI 1.89–5.29) and an HIV positive status (HR 2.28, 95% CI 1.14–4.56). This study provides a comprehensive description of ICU patient characteristics, treatment patterns, and outcomes in Uganda. It not only adds to the global body of knowledge on ICU care in resource-limited settings but also serves as a foundation for future research and policy initiatives aimed at optimizing ICU care in Sub-Saharan Africa.

## Introduction

In high-income countries (HICs), advancements in critical care have consistently yielded decreasing Intensive Care Unit (ICU) mortality rates, even as the population ages and contends with intricate health issues^1–3^. Contrarily, Low and Lower-Middle Income Countries (LMICs) grapple with an increased mortality burden, especially with conditions like sepsis, trauma, and obstetric complications, which disproportionately impact on ICU mortality in these settings when compared to HICs^4–9^.

Distinctly in LMICs, the typical ICU cases are young, male, post-operative patients, yet they face an estimated mortality rate that’s nearly fourfold higher than their counterparts in HICs^7,10–12^. This discrepancy was markedly evident during the Coronavirus induced disease 2019 (COVID-19) pandemic in African nations^13^. Postulations attribute this elevated mortality rate to a combination of patient-centric factors—predominantly the acuteness of illness exacerbated by delayed or constrained access to critical care—and systemic factors like inadequate critical care infrastructure^14,15^. Specific to Uganda, the prevailing scarceness of ICU resources, evidenced by very low metrics such as ICU bed density (one bed per million population) and skilled healthcare professionals, has been underscored^7,16^.

Research on this matter, especially from Africa, is predominantly sourced from isolated centers or predominantly urban, academic-affiliated establishments^14,17^, thus failing to capture a holistic national perspective that accounts for the inherent heterogeneity between urban and rural, and public and private healthcare settings. Notably, the distinctiveness of case-mixes between different ICU settings and regions may yield diverse outcomes owing to the varied capacities to implement essential care processes. Additionally, with the increasing burden of non-communicable diseases in LMICs, their possible contribution to critical illness is still underexplored^17^.

Consequently, this study aimed to describe patient characteristics and subsequent 28 day outcomes including mortality rates, ICU durations, and organ dysfunctions across all functional ICUs in Uganda.

## Method

### Study design and setting

**Figure 1:**
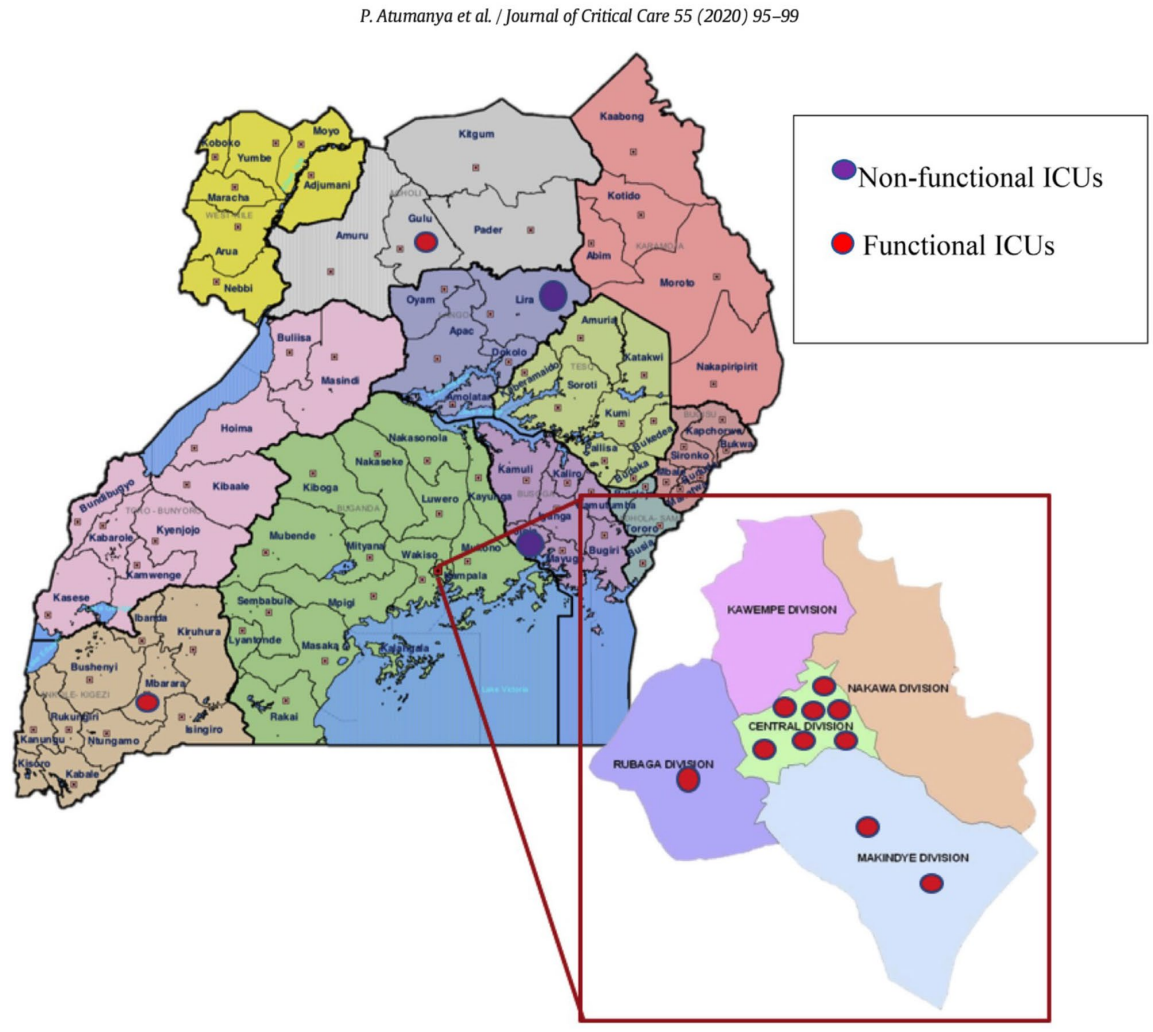
Geographical location of Uganda’s ICUs that recruited the study population.

This study is a multicenter, prospective, observational cohort investigation conducted across 12 operational Intensive Care Units (ICUs) in Uganda, from January to April 2018. The characteristics of these ICUs have been detailed previously (see Fig. 1)^16^. A “functional ICU” was defined as a designated area equipped for admitting critically ill patients, capable of providing mechanical ventilation, airway suction, continuous monitoring, and bedside nursing care. According to prior research, approximately 83% of these ICUs are located within Kampala, the capital city^16^. The same study revealed that 75% of the ICUs were privately operated (both for-profit and nonprofit organizations), 42% were affiliated with university hospitals, and 75% were overseen by anesthesiologist-led ICU directors. Notably, over 90% admitted both adult and pediatric patients with medical-surgical needs. Regarding operational models, 33% of the ICUs followed an open-ICU approach, with a nurse-patient ratio ranging from 1:2 to 1:4. This study adheres to the STROBE (Strengthening the Reporting of Observational Studies in Epidemiology) guidelines.

### Participants

The study included all patients admitted to the participating ICUs within the specified study timeframe. Exclusions were made for patients with incomplete daily medical records or those assessed by attending physicians as not requiring ICU-level care. Patients transferred to another unit were followed for the primary outcome only.

### Data collection

Recruitment spanned three months at each ICU, from 8th January to 1st April 2018, with follow-ups extending to 30th April for the last enrolled patients. Data was collected from daily observation and medical records within the initial 24 h of admission, utilizing pretested, paper-based case report forms. Collected data included demographic information, severity of illness, source of admission, reasons for ICU admission, medical or surgical admission status, comorbidities, readmission status, vital signs in the first 24 h, interventions and treatments during the ICU stay, and 28 day ICU patient outcomes. Laboratory and arterial blood gas results within the first 24 h were also collected when available. The severity of critical illness in the first 24 h was assessed using the National Early Warning Score-2 (NEWS-2) and the quick Sequential Organ Function Assessment (qSOFA), selected for their reliance on vital signs and Qsofa’s additional utility as a mortality prognostic tool^18^.

Patients were monitored for 28 days or until ICU discharge or death, whichever occurred first. Patients in the ICU beyond 28 days were considered survivors. Mortality was confirmed by attending physicians.

Based on previous studies^7,19^, the expected nationwide ICU mortality rate was approximately 30%. Using the Kish-Leslie formula (1965), a 5% precision level, and a 95% confidence interval, we estimated a sample size of 320. To account for potential follow-up losses, we adjusted this figure to 352 participants. For analyzing ICU mortality correlations, we posited that surgical admissions would have a 34.0% mortality rate versus 50.0% for medical admissions, based on preliminary data. With an 80% power and a 10% anticipated non-response rate, we calculated a sample size of 165 participants for this analysis, choosing the larger sample size for the primary outcome assessment.

### Statistical analysis

The primary outcome was all-cause 28 day ICU mortality. Secondary outcomes assessed included the length of ICU stay, required organ support/interventions, and mortality-associated factors. Data was processed using Epidata software (version 3.1) and analyzed in STATA 12.0. Continuous variables were summarized as means (standard deviation) or medians (interquartile range), and categorical data as percentages. Mortality rates were calculated for each ICU location. Patient outcomes were dichotomized into survivors and non-survivors, with Kaplan–Meier survival curves generated and evaluated via the Log-Rank test with the assumption that patients discharge alive before 28 days were alive at 28 days. The chi-square test and Fisher’s exact test were applied for categorical comparisons, with the Student’s t-test or Mann–Whitney test for continuous variables. Multivariate Cox regression models were employed to identify independent risk factors for ICU mortality, selecting variables based on biological plausibility or a bivariate p-value < 0.2, considering a two-tailed p-value < 0.05 as statistically significant.

### Ethical approval

Ethical approval was granted by the Makerere University School of Medicine Research and Ethics Committee (SOMREC #REF 2018–004). The study was registered at clinicaltrials.gov (#NCT03511742) and conducted in accordance with the Declaration of Helsinki. Informed consent was obtained from all participants and/or their legal guardian(s) where the patients were unable to give informed consent.

## Results

### Patient enrolment and distribution

**Figure 2:**
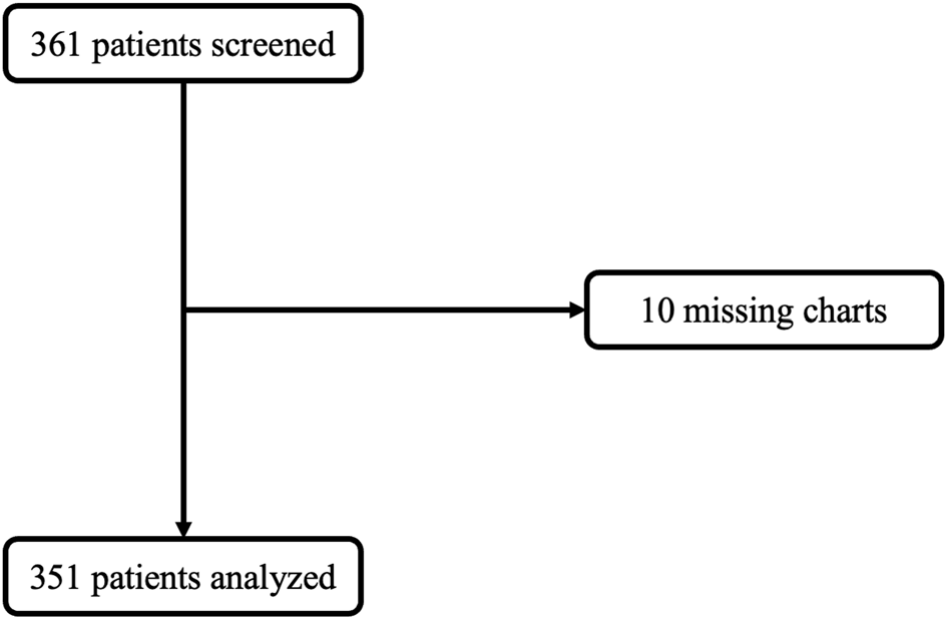
Patient flow chart.

In this study, 361 patients across 12 functional Intensive Care Units (ICUs) in Uganda were initially screened, with 10 exclusions due to incomplete patient monitoring charts, yielding a final sample of 351 patients (Fig. 2). The majority (84%) of these patients originated from urban centers, with 25.1% admitted to ICUs within public hospitals **(**see Fig. 1 for the geographical distribution of Uganda’s ICUs**)**.

### Baseline characteristics on ICU admission

The cohort comprised 10% foreigners and 42% females, with a mean age of 39.2 years (SD = 24.1 years). Pediatric patients (< 18 years) constituted 21.1% of the sample. Age distribution peaked within the 25–59 year range (50%), followed by the elderly (≥ 60 years, 22%) and children under 15 years (19.4%). Missing data included mean arterial pressure (MAP) values for 27 patients (7.7%) and Glasgow Coma Scale (GCS) scores for eight patients (2.3%). The median qSOFA score was 2 (IQR, 1–3), with 42.5% of patients scoring ≥ 2 and 20.3% having a NEWS-2 score < 7. Notably, 214 (61%) had at least one comorbidity, such as hypertension, diabetes, or chronic kidney disease, and 10% were HIV positive, though 53% had an unknown HIV status upon admission. The primary sources of ICU admissions were the operating room (112, 32%) and general wards (97, 27.7%), with 56% of admissions related to surgery. The most common reasons for ICU admission included postoperative care (42.9%), altered mental status (34.5%), and acute respiratory failure (32.8%) **(**Table 1**).**

**Table 1:** Baseline characteristics of patients admitted to 12 ICUs in Uganda from January 2018 to April 2018.

Characteristics	All (351), N (%)	Survivors (261), N (%)	Non-survivors (90), N (%)	P value*
Age, mean (SD), Years	39.2 ± 24.1	38.1 ± 1.5	42.3 ± 2.3	0.156
Pediatric (< 18 years), n (%)	74 (21.1)	59 (22.6)	15 (16.7)	0.234
Sex; female, n (%)	146 (41.6)	107 (41.0)	39 (43.3)	0.698
Nationality; Ugandan, n (%)	311 (89.1)	234 (89.7)	77 (85.5)	0.575
Admission source, n (%)^a^				
Emergency department	79 (22.6)	66 (25.4)	13 (14.4)	
Hospital ward	97 (27.7)	62 (23.8)	35 (38.9)	
Operating room	112 (32.0)	93 (35.8)	19 (21.1)	
Referral	62 (17.7)	39 (15.0)	23 (25.6)	**0.001**
Admission status, n (%)				
Medical	154 (43.9)	106 (40.6)	48 (53.3)	
Surgical	182 (51.8)	148 (56.7)	34 (37.8)	**0.002**
Comorbidities, n (%)				
Hypertension	104 (48.6)	73 (46.2)	31 (55.4)	
Diabetes type 2	48 (22.4)	35 (22.2)	13 (23.2)	
Chronic kidney disease	26 (12.1)	20 (12.7)	06 (10.7)	
HIV	34 (9.7)	20 (7.7)	14 (15.6)	0.107
Indication for ICU admission, n (%)				
Altered mental status	121 (34.5)	84 (32.2)	53 (41.1)	0.124
Acute respiratory failure	115 (32.8)	68 (26.1)	47 (52.2)	** < 0.001**
Sepsis/septic shock	76 (21.7)	40 (15.3)	36 (40.0)	** < 0.001**
Postoperative care (n = 150)^b^				
Neurosurgery	22 (14.7)	21 (16.8)	1 (4.0)	
Cardiac surgery	43 (28.7)	36 (28.8)	7 (28.0)	
Others	85 (56.6)	68 (54.4)	17 (68.0)	0.218
Readmission, n (%)	10 (2.8)	05 (1.9)	05 (5.6)	0.132
MAP (mmHg); median (IQR)^c^	81 (62–107)	84 (65–109)	68 (52–99)	**0.002**
GCS, median(IQR)^d^	12 (7–15)	14 (8–15)	6 (3–10)	** < 0.001**
NEWS-2 Score ≥ 7, n (%)	193 (79.7)	142 (75.9)	51 (92.7)	**0.023**
qSOFA score ≥ 2, n (%)^e^	169 (57.5)	118 (52.7)	51 (72.9)	**0.011**
ICU Length of stay; median (IQR)	3 (1–7)	3 (2–7)	3 (1–7)	0.539

*HIV* human immunodeficiency virus, *GCS* Glasgow Coma Scale, *IQR* interquartile range, *NEWS*-2 National Early Warning Score 2, *qSOFA* quick sequential organ function assessment.

^a^One participant missing admission source. ^b^One patient had missing data for postoperative indication. ^c^Only 324 patients had complete values for mean arterial pressure, ^d^Only 343 patients had complete values for Glasgow Coma Scale. ^e^Only 294 had complete data to calculate qSOFA.

Significant values are in bold.

### Interventions and treatments during ICU stay

Nearly half of the patients (116, 45%) received mechanical ventilation and analgo-sedation, with or without paralysis. About a third required vasopressor/inotropic support, and (24, 6.8%) underwent renal replacement therapy. Antibiotics were administered to 87.5% of the patient population **(**Table 2**).**

**Table 2:** ICU interventions and Organ support for patients admitted to 12 ICUs in Uganda from January 2018 to April 2018.

Intervention	All (351), N (%)	Survivors (261), N (%)	Non-survivors (90), N (%)	p*
Mechanical ventilation	161 (45.0)	89 (55.3)	72 (44.7)	**0.001**
Analgosedation/paralysis	194 (55.3)	131 (67.5)	63 (32.5)	**0.001**
Inotropes/vasopressors	109 (31.1)	58 (53.2)	51 (46.8)	** < 0.001**
RRT	24 (6.8)	11 (45.8)	13 (54.2)	**0.001**
Blood transfusion	131 (37.3)	90 (68.7)	41 (31.3)	0.157
Antibiotic use	307 (87.5)	227 (73.9)	80 (26.1)	0.890

*RRT* renal replacement therapy.

p* values calculated using chi square or Mann–Whitney U test.

Significant values are in bold.

### Patient outcomes and survival analysis

The 28 day crude ICU mortality rate was 25.6% (95% CI 21.3–30.4), with a higher mortality observed in Private-Not-For-Profit (PNFP) ICUs compared to private and public hospitals (33.0% vs 26.2% vs 17.2%; p = 0.035). Non-survivors were more likely to have been referred from another hospital or transferred from a ward, to have medical diagnoses, to suffer from sepsis or acute respiratory failure, and to have lower mean arterial pressures and Glasgow Coma Scale scores. They were also more likely to receive organ support, such as mechanical ventilation (80% vs 34%; p = 0.001), sedation/paralysis (70% vs 50%; p = 0.001), inotropic/vasopressor support (57% vs 22%; p < 0.001), and renal replacement therapy (14% vs 4%; p = 0.001) Table 2.

**Figure 3:**
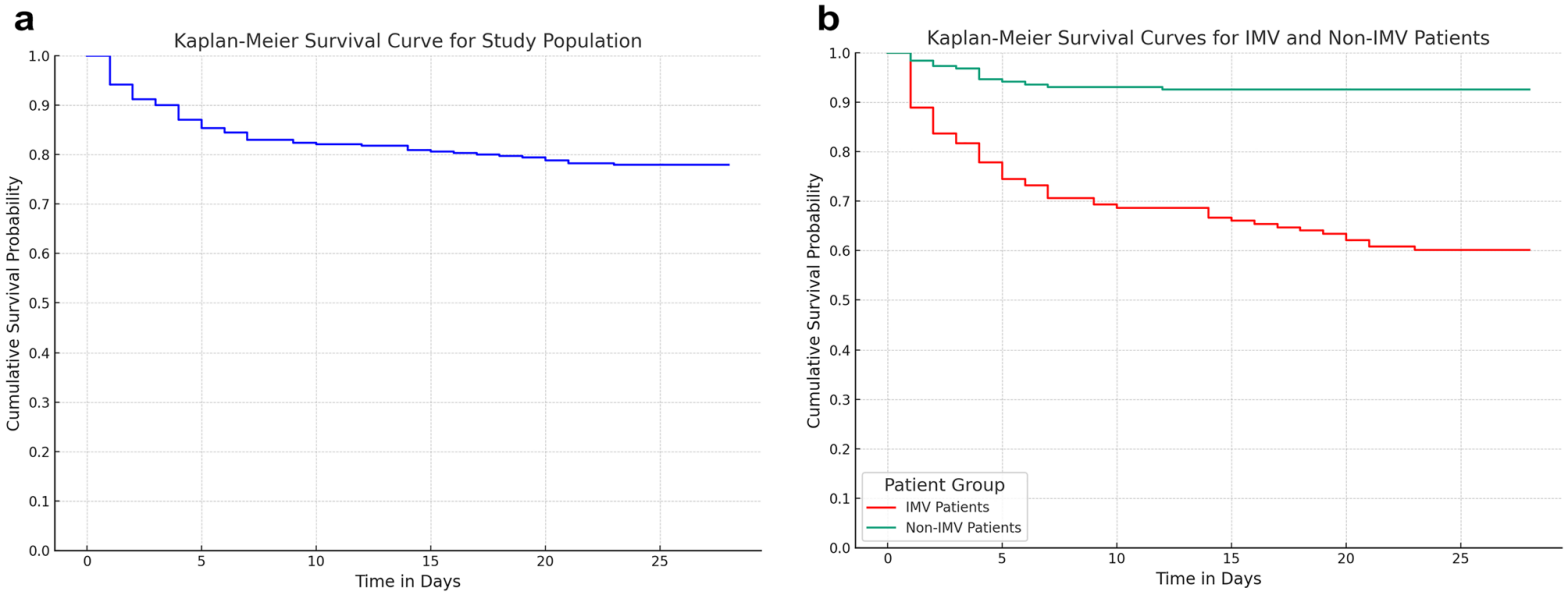
Kaplan Meier Curves: 3a shows the entire study population while 3b shows comparison between mechanically ventilated patients and non-mechanically ventilated patients.

The median length of ICU stay was 3 days, with a mean of five days, showing no significant difference between survivors and non-survivors. The Kaplan–Meier survival curve indicated that the probability of death was highest within the first 10 days of admission but decreased by day 20, stabilizing up to day 28. Patients requiring mechanical ventilation showed significantly lower survival probabilities from the point of admission (p < 0.001) **(**Fig. 3**)**.

### Factors associated with mortality

Key risk factors for ICU mortality included age ≥ 45 years (adjusted Hazard Ratio [aHR] = 10.31, 95% CI 1.1–99.7), the necessity of mechanical ventilation (aHR = 11.09, 95% CI 2.53–48.59), the requirement for vasopressor/inotropic support (aHR = 1.89, 95% CI 1.12–5.29), and a positive HIV status (aHR = 2.28, 95% CI 1.14–4.56). The source of hospital funding did not significantly influence ICU mortality rates Table 3.

**Table 3:** Factors associated with Mortality of 351 patients admitted to 12 ICUS in Uganda from January 2018 to April 2018 at bivariate and multivariate analysis.

Characteristic	Unadjusted HR, 95% CI	p valve	Adjusted HR, 95% CI	p* value
Age ≥ 45 years	2.04 (0.73–5.68)	0.172	10.31 (1.1–99.7)	**0.044**
HIV positive	2.24 (1.18–4.23)	0.013	2.28 (1.14–4.56)	**0.034**
Mechanical ventilation	3.18 (1.88–5.36)	< 0.001	11.09 (2.53–48.59)	**0.001**
Inotropes/vasopressors	2.58 (1.69–3.93)	< 0.001	1.89 (1.12–5.29)	** < 0.001**
Admission diagnosis				
Respiratory disease**	1.61 (1.03–2.52)	0.036	0.09 (0.02–0.49)	**0.005**
Hospital funding type***				
PNFP	2.04 (1.07–3.91)	0.031	2.35 (0.69–7.90)	0.167
Private	1.61 (0.85–3.05)	0.144	1.33 (0.24–7.44)	0.739

*HIV* human immunodeficiency virus, *RPT* renal replacement therapy, *GCS* Glasgow Coma Scale, *PNFP* private-not-for-profit.

p* used cox regression analysis. **Non-respiratory disease at admission was the reference point. ***Public Hospital ICU was the reference point.

Significant values are in bold.

## Discussion

This pioneering multicenter, prospective study, the first of its magnitude conducted in Uganda’s pre-COVID-19 landscape, meticulously examines the demographics and outcomes of 351 ICU patients, revealing a significant urban bias in ICU admissions, with a quarter directed to public hospitals. The cohort, characterized by a notable youthfulness and a significant pediatric representation (21%), predominantly comprised surgical patients, with nearly two-thirds presenting at least one comorbidity. The findings underscore a pressing concern: the potential overutilization of critical care resources, highlighted by a substantial portion of patients with low severity scores (qSOFA < 2 and NEWS-2 < 7) potentially not necessitating ICU care. This study serves as a critical baseline, offering insight into the dynamics of ICU care within the region, and establishes a pivotal reference for future studies, particularly those assessing the impacts of the COVID-19 pandemic.

The study’s timing and scope provide a vital baseline for understanding ICU care dynamics in the region and serve as a reference point for future comparative analyses, especially in the wake of the COVID-19 pandemic. The urban-centric (84%) distribution of ICU admissions, and predominantly in private-funded hospitals (75%), raises significant concerns about healthcare accessibility in rural areas yet eight out of 10 of the population lives in rural areas^20^. With only one percent of Uganda’s population earning more than 3000 USD per annum^21^, this disparity is further exacerbated by the lack of a national health insurance scheme in Uganda, leading to inequitable access based on socioeconomic status^22^. The findings call for urgent policy interventions to bridge this urban–rural healthcare divide.

We noted a younger average age than the aggregated age in HICs (39.2 vs. 60–70 years)^1–3^, but similar to that in other similar LMICs and prior studies done in Uganda^6–9,23,24^. This is consistent with Uganda’s demographic profile that has at least 75% of its population under 30 years^20^. Indeed, we had a notable pediatric (21.1%) because at the time of this study, there were no dedicated pediatric ICUs. Sub-Saharan Africa (SSA) probably has the highest burden of critical illness^4,5^, however, we found four in ten patients admitted to our ICUs probably did not need ICU admission. For example, one of the hospitals had an admission policy that required all post-tonsillectomy (a day case surgery) patients to be admitted to the general ICU. This suggests the need for more refined ICU admission criteria and improved triage processes^25,26^. The predominance of surgical cases, especially in public ICUs, and a high prevalence of comorbidities, including HIV, paints a detailed picture of the rising burden non-communicable diseases on a background of a high burden of infectious diseases posing significant health challenges^27^. The high rate of antibiotic usage observed in the study is a red flag for potential antibiotic resistance, a growing global health concern^28^. This underscores the need for stringent antibiotic stewardship programs, especially in resource-limited settings where the burden of infectious diseases is high^27^. The significant number of missing values in key clinical parameters like MAP and GCS raises concerns about the quality of clinical documentation and potential resource constraints in ICU settings. This gap in data could have profound implications for patient monitoring, outcome predictions, and overall ICU management strategies.

Our study revealed notable differences in patient profiles between private and publicly funded hospitals. Private hospitals, predominantly accessed by those of higher socio-economic status, had a higher proportion of non-communicable diseases including HIV patients, and as such medical admissions. This is in line with global trends where socio-economic status often dictates the nature of health challenges faced by individuals^1,2,29^. Conversely, publicly funded ICUs primarily admitted young males with neuro-trauma, reflecting perhaps the epidemiology of trauma in Uganda^30^. This pattern highlights the urgent need for targeted interventions in trauma care and prevention, especially among the young male population. Over half of the patients had an unknown HIV status at admission, which could point to systemic gaps in HIV testing and documentation in critical care settings. The proportion of HIV-positive patients in our study (9.7%) was significantly higher than in HICs (0.7%), but much lower compared to other regions in Sub-Saharan Africa, such as Zambia (71%)^9,31^. This discrepancy might be attributable to an inherent bias in ICU admissions concerning HIV patients, potentially due to anticipated poor outcomes^32^. The increased risk of death in HIV-positive patients that we found, echoes findings from South African studies, and underscores the need for integrating HIV status into ICU care protocols and improving HIV testing and documentation practices.

The study’s mortality analysis revealed a 28 day ICU mortality rate of 25.6%, which is notably lower than previous studies conducted in similar settings including Uganda^7,23,24,33^. In addition, a studies done in East African neighbors, showed a significantly higher ICU mortality (25.6 vs 45%)^6,8^. Predominantly surgical ICU admissions may also lead to less ICU mortality as was also seen in Malawian study that had over 70% of the ICU population being surgical and had an ICU mortality (23.6%) similar to our study^34^. This could be attributed to the inclusion of less critically ill patients, as indicated by the significant number of admissions with lower severity scores. The highest probability of death within the first 10 days of ICU admission is not only similar to prior studies^23^ but also underscores the critical nature of early ICU care. Interestingly, publicly funded ICUs, despite being the least resourced^16^, showed the lowest mortality rate suggesting that factors beyond resource availability, such as patient demographics, admission criteria and organizational factors, play a vital role in outcomes^35^. There was no significant association with mortality regarding hospital-funding type however, age, HIV positive status and requirement of ICU organ support was significantly associated with ICU mortality.

The study’s strengths include its multicenter, prospective design, providing a comprehensive view of ICU care across different hospital settings in Uganda. This enhances the generalizability and applicability of the findings, making it a valuable resource for policymakers and health administrators. However, limitations such as the short study duration, lack of daily patient-level clinical data, and reliance on patient charts leading to some data gaps, must be acknowledged. These limitations highlight areas for improvement in future research, particularly in enhancing data collection methods and extending study durations to capture more longitudinal patient data.

## Conclusion

This landmark study provides a comprehensive overview of ICU patient characteristics, treatment patterns, and outcomes in Uganda. Its findings are crucial for informing ICU care strategies, guiding resource allocation, and shaping policies to improve critical care in LMICs. The study not only adds to the global body of knowledge on ICU care in resource-limited settings but also serves as a foundation for future research and policy initiatives aimed at optimizing ICU care in Sub-Saharan Africa.

## Data Availability

The datasets used and/or analyzed during the current study are available from the corresponding author on reasonable request.

## Abbreviations

HICs: High income countries
ICU: Intensive care unit
LMICs: Low- and lower-middle income countries
COVID-19: Coronavirus induced disease 2019
NEWS-2: National Early Warning Score-2
QSOFA: Quick sequential organ function assessment
SOMREC: School of Medicine Research and Ethics Committee
SD: Standard deviation
IQR: Interquartile range
LOS: Length of stay
HIV: Human immunodeficiency virus
GCS: Glasgow Coma Scale
APACHE II: Acute Physiologic Assessment and Chronic Health Evaluation II
